# Supplementary figures and images for: Schistosoma mansoni infection induces plasmablast and plasma cell death in the bone marrow and accelerates the decline of host vaccine responses
Source: PLoS Pathog. 2022 Feb 14;18(2):e1010327. doi: 10.1371/journal.ppat.1010327 (PMC8893680; doi:10.1371/journal.ppat.1010327)

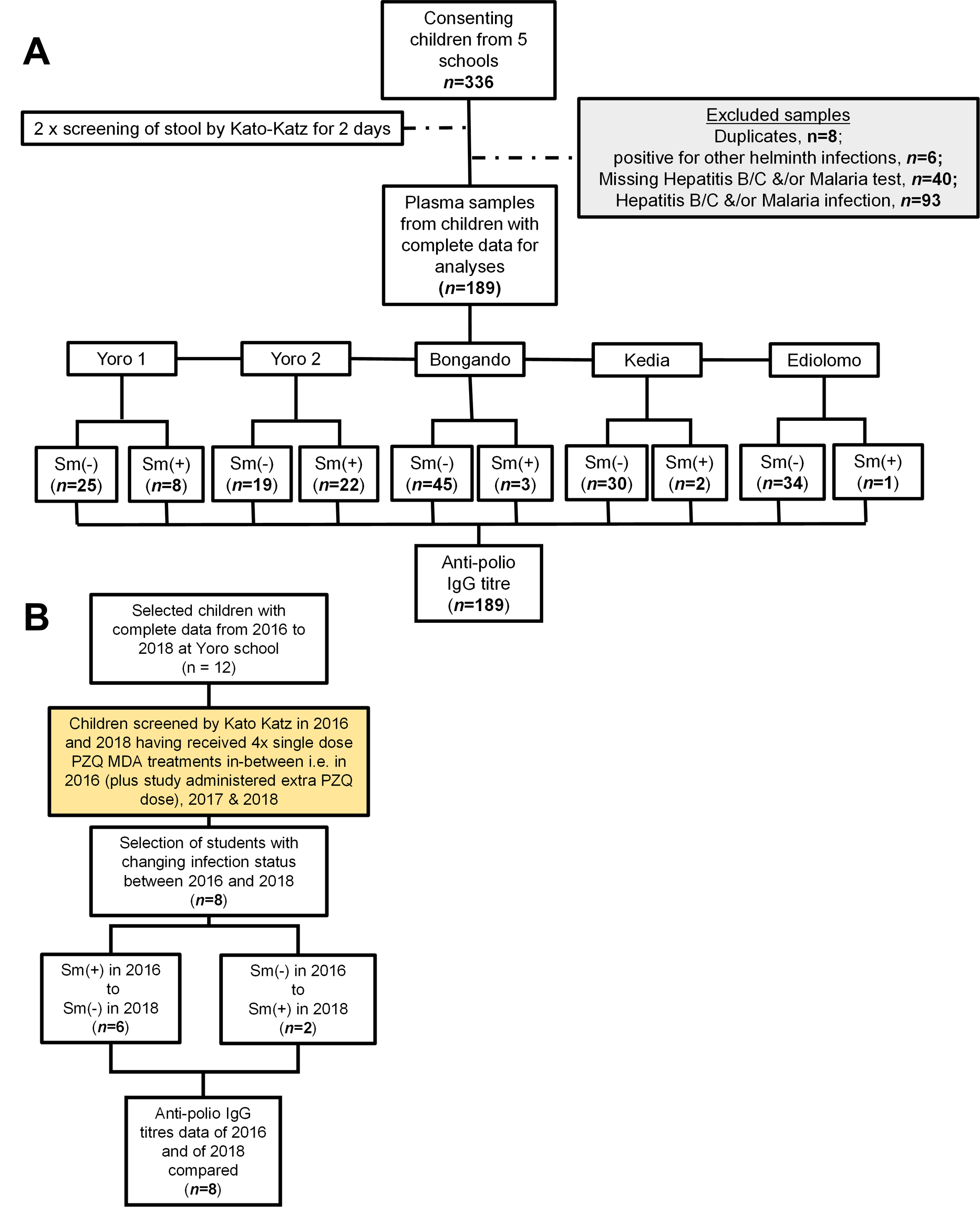

Supplement: S1 Fig — (A) A total of 336 consenting/assenting children, with consent from legal guardians, were enrolled from all schools combined. All children completed questionnaires and had 2 stool samples collected on two separate days for S. mansoni egg detection by the Kato-Katz technique. Plasma was collected only for children without missing data nor infections such as other helminths, malaria, and hepatitis B and/or C. A battery of exams and diagnostic tests were performed on consenting participants and only samples from patients with no missing data for stool examination by Kato Katz, rapid diagnostic testing of Hepatitis B and C viruses and microscopical screening of malaria parasite in blood smears were further used in the present study, as previously described. Finally, 189 children had their plasma samples analysed for anti-poliovirus IgG titres. (B) Samples of children with complete questionnaire data showing complete annual PZQ treatment, and complete anti-poliovirus IgG titre analyses from two studies conducted three years apart were selected. Initial data was collected in the first study conducted in the year 2016 followed by data collection for the same children in a second study in the year 2018. A total of 8 children were identified. These children were treated for schistosomiasis with a dose 40mg/kg of PZQ once in March in the years 2016, 2017 and 2018 under the National Program for the Control of Schistosomiasis and Soil transmitted Helminthiasis of the Ministry of Public Health in Cameroon. Additionally, in 2016, all study participants were treated with a PZQ dose despite infection status, thus totalling 4 doses during the 3-year study period. (TIF) [file ppat.1010327.s001.tif]

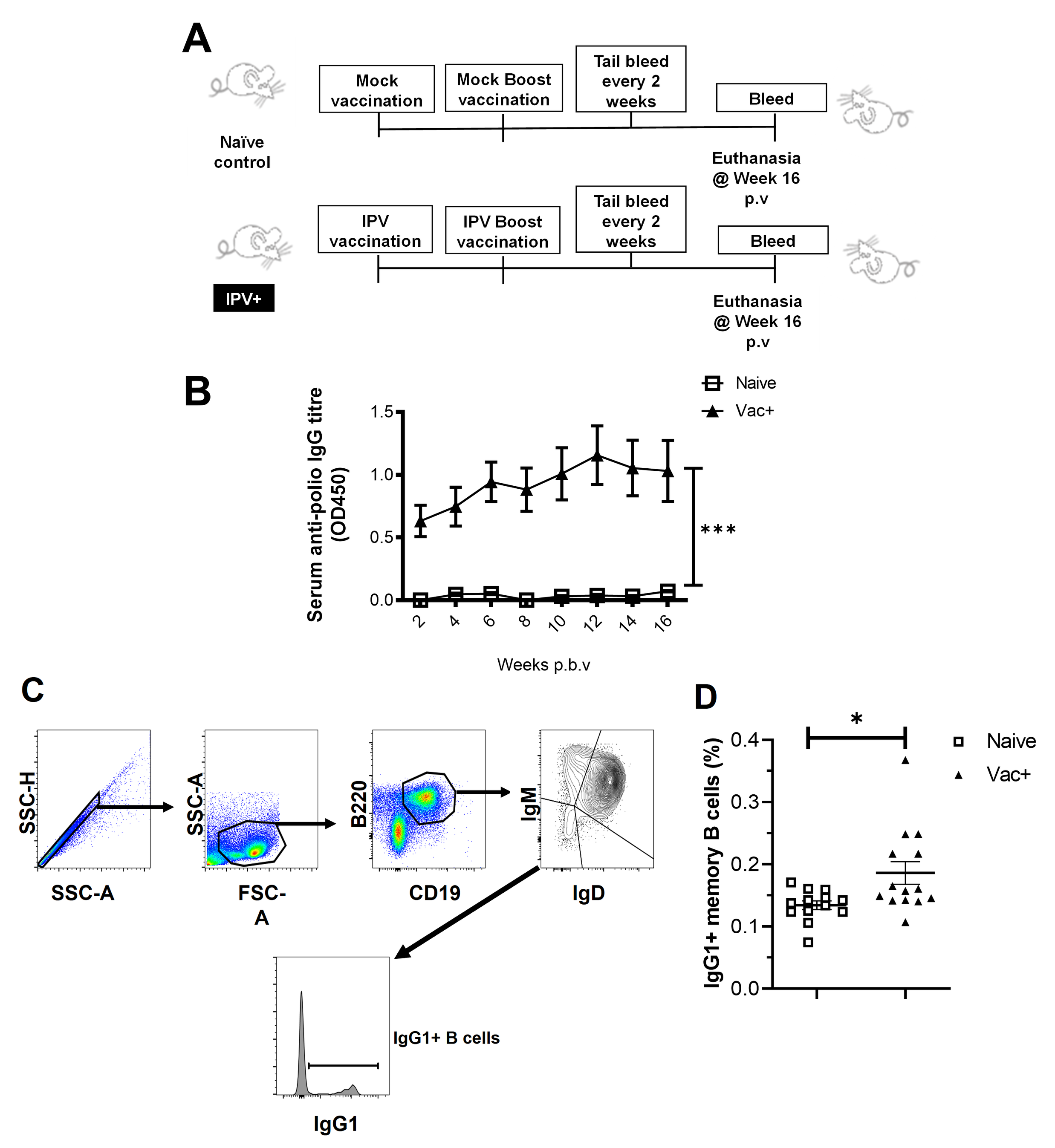

Supplement: S2 Fig — (A) Experimental design: BALB/c mice (6 to 8 weeks old and acclimatized for 1 week before procedure) were mock vaccinated (naive) or injected subcutaneously with a commercialized hexavalent vaccine, hexavalent (DTPa-hepB-IPV-Hib) vaccine, at 2 sites i.e., 150ul on the neck and 150ul on the abdomen at day 0 and day 30. Blood was collected from tail vein (approximately 50ul) every two weeks until day 142 (week 16 after second vaccine dose). The animals were sacrificed at week 16, terminally bled and their spleens were collected. (B) Serum isolated from blood samples was probed by ELISA for anti-Polio virus IgG titres over time. C. Gating strategy for class switched IgG1 memory B cell. D. IgG1 memory B cells in vaccinated mice in comparison to naïve mice. Data are expressed as mean ± S.E.M and representative of 2 independent experiments (n = 13–14 mice per group in each experiment); Data was analysed by two-way ANOVA followed by FDR corrected multiple comparisons; ns, p> 0.05; * p< 0.05, ** p< 0.001, *** p< 0.0001; Vac, hexavalent (DTPa-hepB-IPV-Hib) vaccine; p.v, post-vaccination. (TIF) [file ppat.1010327.s002.tif]

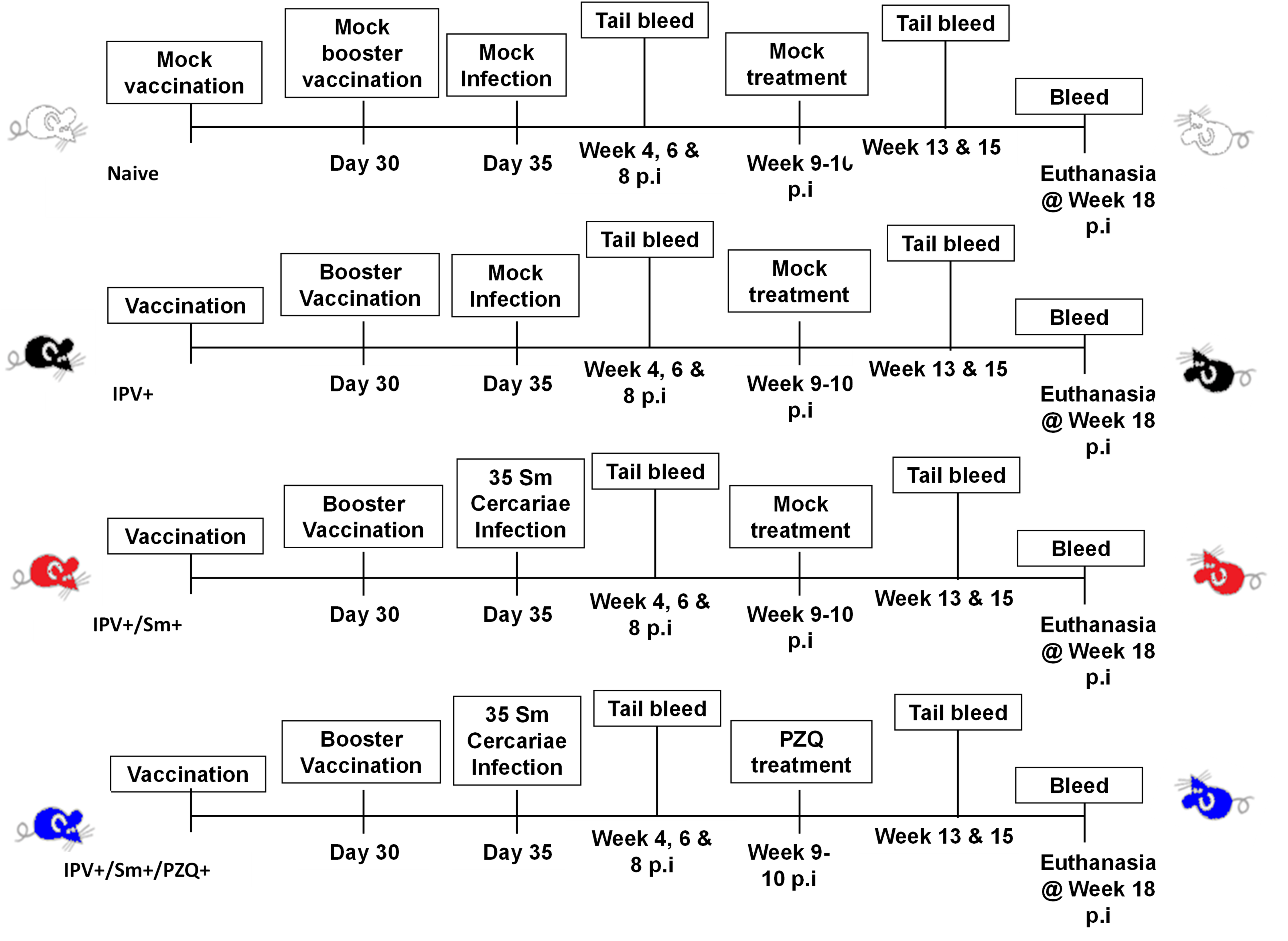

Supplement: S3 Fig — BALB/c mice (6–8 weeks old) were separated into 4 experimental groups. (A) was injected with phosphate buffered saline (PBS). (B-D) Mice were injected subcutaneously with a commercialized hexavalent vaccine, hexavalent (DTPa-hepB-IPV-Hib) vaccine, at 2 sites i.e., 150ul on the neck and 100ul on the abdomen, at day 0 and day 30. Five days after the second vaccine dose (day 35), Naïve (A) and IPV-Vaccinated control mice (B) were mock infected with PBS while groups (C) (vaccinated then S. mansoni infected i.e., Vac+Sm+) and (D) (Vaccinated then S. mansoni infected then PZQ treated i.e., Vac+Sm+PZQ+) were percutaneously infected with a low dose of S. mansoni (35 cercariae) to establish a chronic disease course. At the beginning of week 10 after infection (end of week 9 p.i), (A-C) were treated with PBS while (D) was treated with 400mg/kg PZQ once daily for one week. From week 4 to week 8, and from week 13 to week 18 (experimental endpoint) p.i, the animals were bled every 2 weeks from the tail vein and serum was obtained. Animals were monitored for schistosomiasis disease progression (daily weight over time) from day zero of infection. The animals were euthanised at week 18 p.i and cardiac blood and organs (spleen, bone marrow, liver, and gut) were collected for further analyses. Vac, hexavalent (DTPa-hepB-IPV-Hib) vaccine; Sm, S. mansoni; PZQ, praziquantel; p.i, post S. mansoni infection. (TIF) [file ppat.1010327.s003.tif]

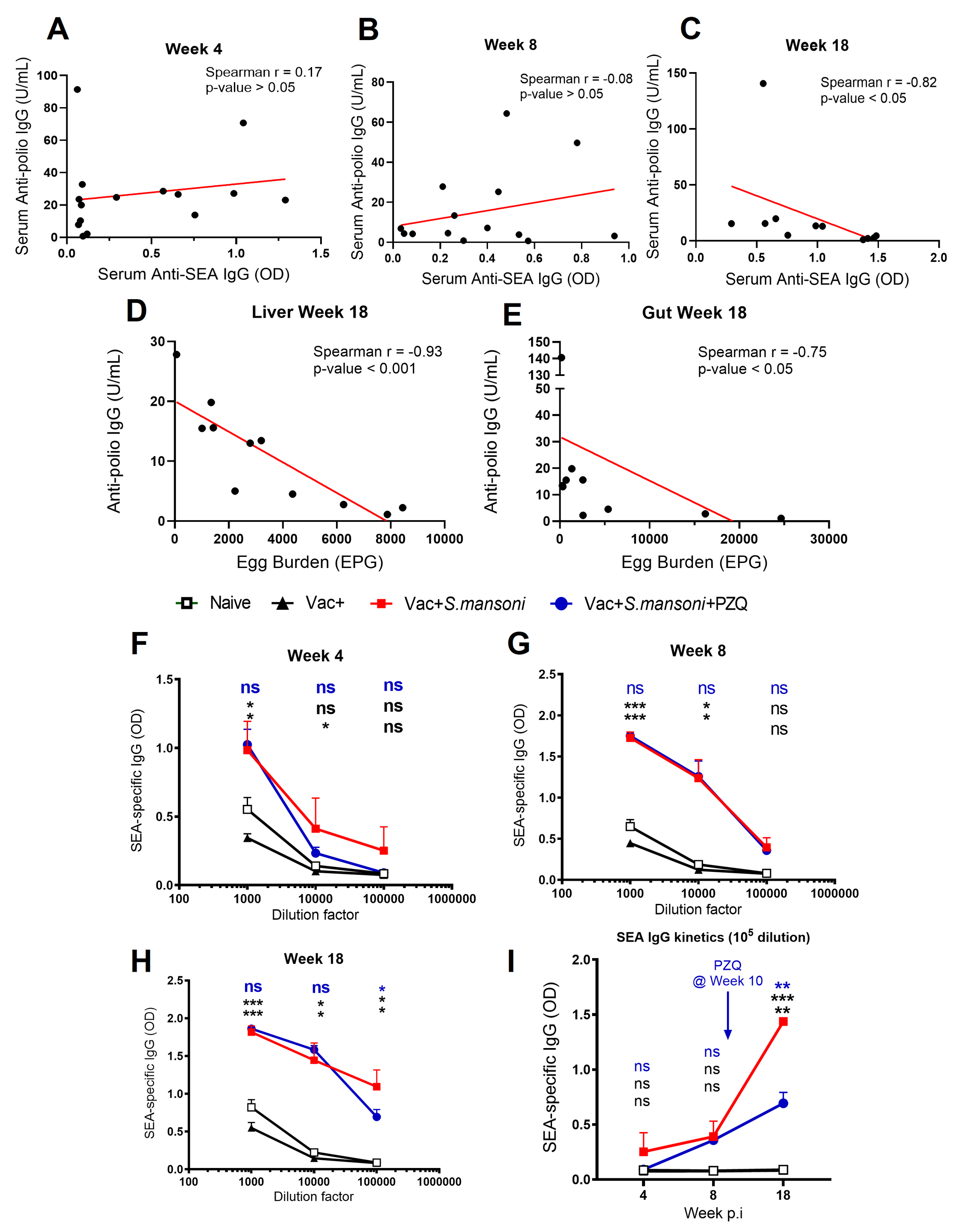

Supplement: S4 Fig — (A) Anti-polio IgG titres vs anti-SEA IgG titres at week 4 p.i. (B) Anti-polio IgG titres vs anti-SEA IgG titres at week 8 p.i. (C) Anti-polio IgG titres vs anti-SEA IgG titres at week 18 p.i. (D) Anti-polio IgG titres vs liver EPG at week 18 p.i. (E) Anti-polio IgG titres vs gut EPG at week 18 p.i. (F) SEA-specific antibodies in serum at week 4 p.i. (G) SEA-specific antibodies in serum at week 8 p.i. (H) SEA-specific antibodies in serum at week 18 p.i. (I) Serum anti-SEA IgG titre kinetics response to schistosomiasis infection at week at week 4, 8 and 18. Data are expressed as mean ± S.E.M and representative of 2 independent experiments (n = 6–10 mice per group in each experiment); The Vac+ group is used throughout as reference for statistical comparison. Data was analysed by two-way ANOVA followed by FDR corrected multiple comparisons against the Vac+Sm+ group; ns, p> 0.05; * p< 0.05, ** p< 0.001, *** p< 0.0001; PZQ, praziquantel; p.i, post schistosomiasis infection. (TIF) [file ppat.1010327.s004.tif]

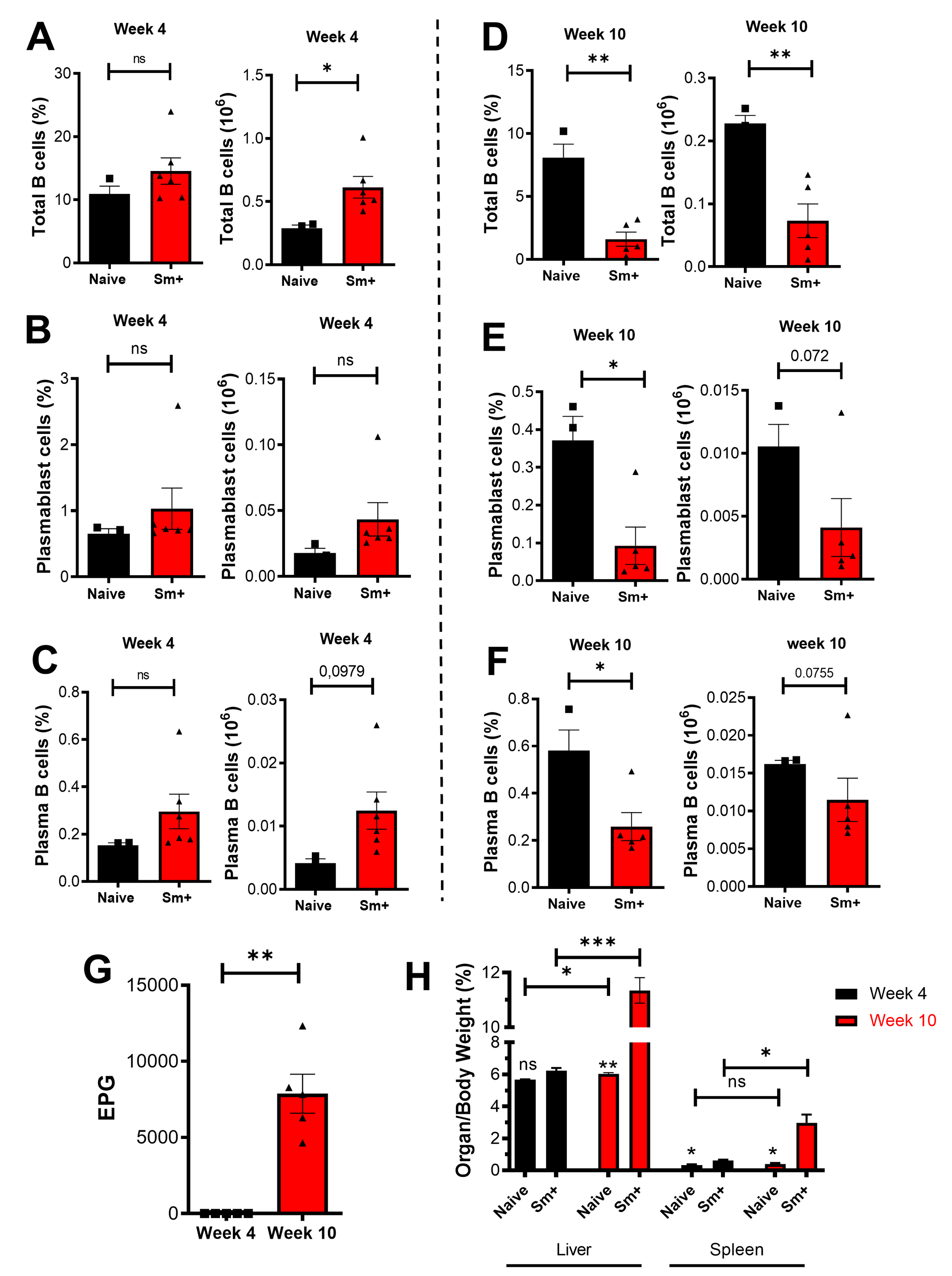

Supplement: S5 Fig — BALB/c mice (6–8 weeks old) were percutaneously infected with a low dose of S. mansoni (35 cercariae) to establish a chronic disease course. Animals were sacrificed at week 4 or week 10 post-infection and B cell populations were quantified in bone marrow and spleen. For week 4 post-infection, (A) Total Bone marrow B cell frequencies and numbers; (B) Total Bone marrow plasmablast frequencies and numbers; (C) Total Bone marrow plasma B cell frequencies and numbers; For week 10 post-infection, (D) Total Bone marrow B cell frequencies and numbers; (E) Total Bone marrow plasmablast frequencies and numbers; (F) Total Bone marrow plasma B cell frequencies and numbers; (G) Liver egg burdens at week 4 and week 10 p.i; (H) Liver and spleen index weights (as a ratio of total body weights) from naïve and infected mice at week 4 vs. week 10 p.i. Data (n = 3–6 mice per group) are expressed as mean ± S.E.M; Data analysed One way ANOVA by followed by the Bonferroni’s multiple comparisons test or by Kruskal wallis followed by the Dunn’s multiple comparisons test; ns, p> 0.05; * p < 0.05, ** p < 0.001, *** p < 0.0001; Sm, S. mansoni; PZQ, praziquantel; p.i, post S. mansoni infection. (TIF) [file ppat.1010327.s005.tif]

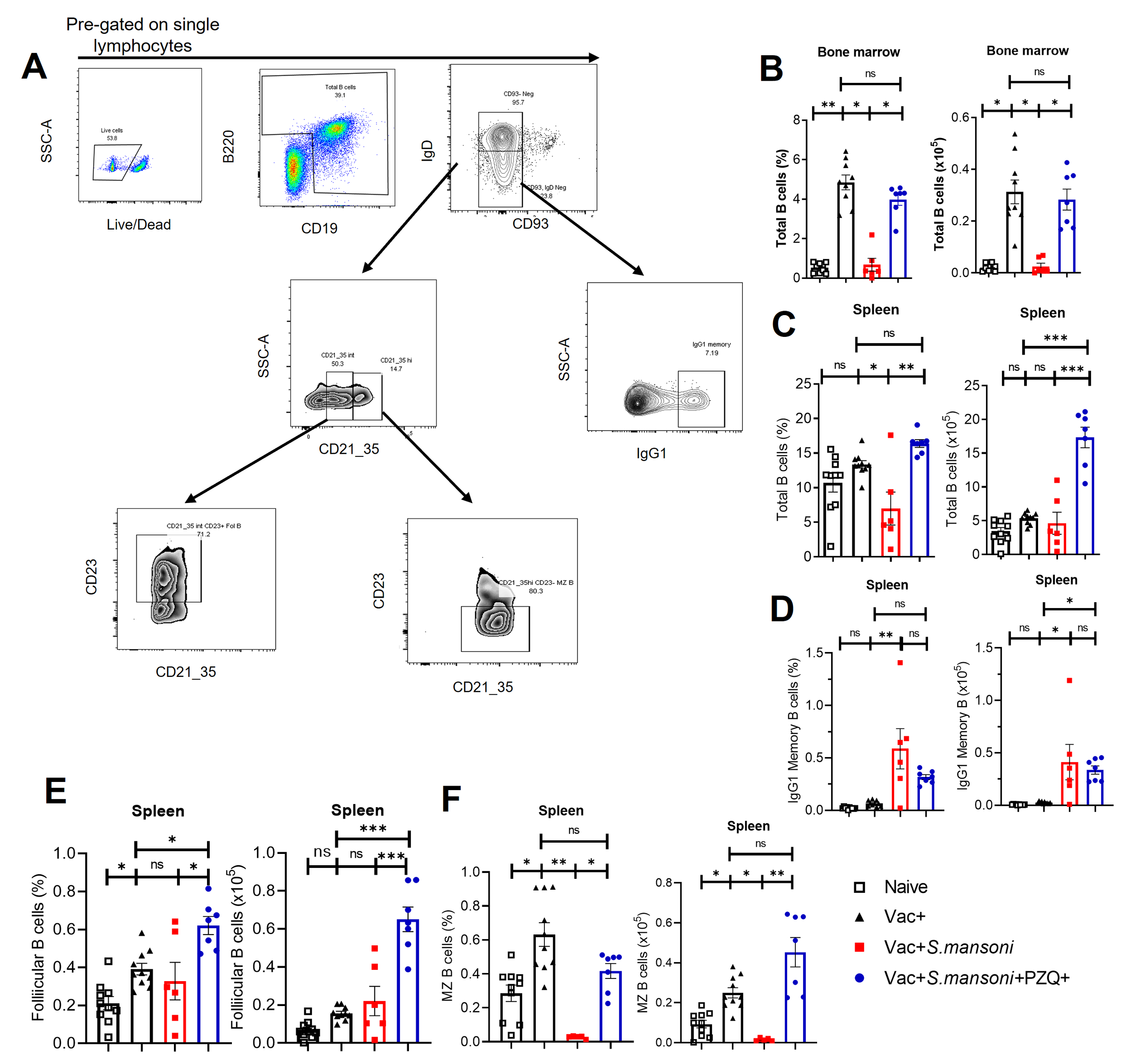

Supplement: S6 Fig — (A) Representative flow cytometry analysis of total B cells from bone marrow and spleen, and IgG1 memory B cells, follicular B cells and MZ B cells from the spleen. (B) Total B cells in the bone marrow. (C) Total B cells in the spleen. (D) IgG1 memory B cells in the spleen. (E) Follicular B cells in the spleen. (F) MZ B cells in the spleen. Data are expressed as mean ± S.E.M and representative of 2 independent experiments (n = 6–10 mice per group in each experiment); Data was analysed by the Shapiro-Wilk test followed by either one way ANOVA with Bonferroni’s multiple comparisons test or by Kruskal Wallis followed by the Dunn’s multiple comparisons test; ns, p> 0.05; * p< 0.05, ** p< 0.001, *** p< 0.0001; Vac, hexavalent (DTPa-hepB-IPV-Hib) vaccine; Sm, S. mansoni; PZQ, praziquantel; SSC, Side scatter; FSC, forward scatter; CD19, cluster of differentiation 19; IgG1, Immunoglobulin G1; IgD, Immunoglobulin D; B220, B cell isoform of 220 kDa; CD93, cluster of differentiation 93; CD21/35, cluster of differentiation 21 and 35; CD23, cluster of differentiation 23. (TIF) [file ppat.1010327.s006.tif]

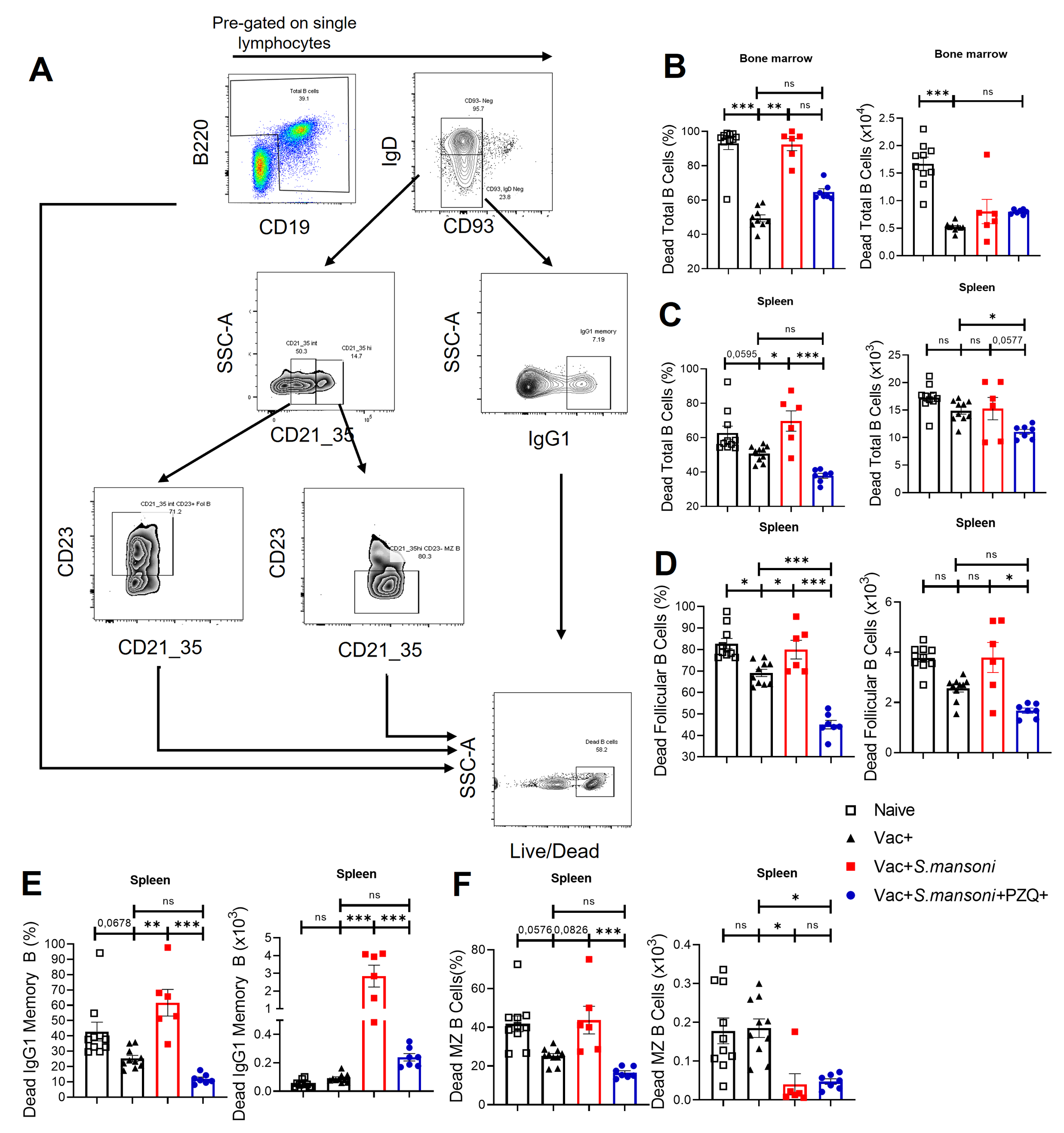

Supplement: S7 Fig — (A) Representative flow cytometry analysis of dead total B cells from bone marrow and spleen, and dead IgG1 memory B cells, follicular B cells and MZ B cells from the spleen. (B) Dead total B cells in the bone marrow. (C) Dead total B cells in the spleen. (D) Dead IgG1 memory B cells in the spleen. (E) Dead follicular B cells in the spleen. (F) Dead MZ B cells in the spleen. Data are expressed as mean ± S.E.M and representative of 2 independent experiments (n = 6–10 mice per group in each experiment); Data was analysed by the Shapiro-Wilk test followed by either one way ANOVA with Bonferroni’s multiple comparisons test or by Kruskal Wallis followed by the Dunn’s multiple comparisons test; ns, p> 0.05; * p< 0.05, ** p< 0.001, *** p< 0.0001; Vac, hexavalent (DTPa-hepB-IPV-Hib) vaccine; Sm, S. mansoni; PZQ, praziquantel; SSC, Side scatter; FSC, forward scatter; CD19, cluster of differentiation 19; IgG1, Immunoglobulin G1; IgD, Immunoglobulin D; B220, B cell isoform of 220 kDa; CD93, cluster of differentiation 93; CD21/35, cluster of differentiation 21 and 35; CD23, cluster of differentiation 23. (TIF) [file ppat.1010327.s007.tif]

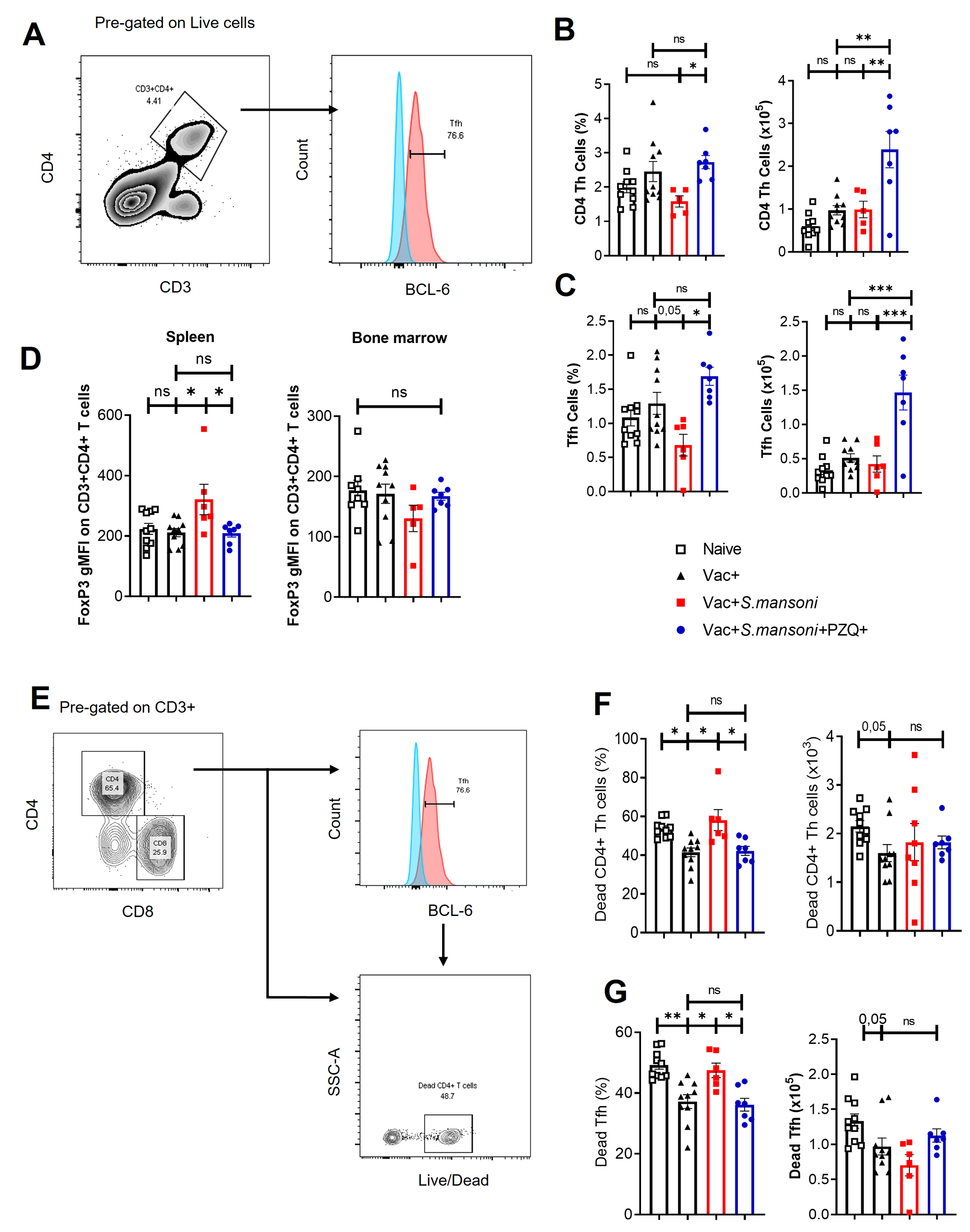

Supplement: S8 Fig — (A) Representative flow cytometry gating strategy for identifying CD3+CD4+ T helper cells and BCL-6+CD3+CD4+follicular T helper cells. (B) CD4+ T helper cells in the spleen. C. BCL-6+CD3+CD4+follicular T cells in the spleen. (D) Foxp3 gMFI expression on CD3+CD4+ T cells in the spleen and bone marrow. (E) Representative flow cytometry gating strategy for identifying dead CD3+CD4+ T helper cells and BCL-6+CD3+CD4+follicular T cells in the spleen. (F) Dead splenic CD3+CD4+ T helper cells. (G) Dead splenic BCL-6+CD3+CD4+follicular T helper cells in the spleen. Data are expressed as mean ± S.E.M and representative of 2 independent experiments (n = 6–10 mice per group in each experiment); Data was analysed by the Shapiro-Wilk test followed by either one way ANOVA with Bonferroni’s multiple comparisons test or by Kruskal Wallis followed by the Dunn’s multiple comparisons test; ns, p> 0.05; * p< 0.05, ** p< 0.001, *** p< 0.0001; Vac, hexavalent (DTPa-hepB-IPV-Hib) vaccine; Sm, S. mansoni; PZQ, praziquantel; SSC, Side scatter; CD3, cluster of differentiation 3; CD4, cluster of differentiation 4; CD8, cluster of differentiation 8; BCL-6, IgG1, Immunoglobulin G1; IgD, Immunoglobulin D; B220, B cell isoform of 220 kDa; CD138, cluster of differentiation 138, BCL-6, B-cell lymphoma 6; Foxp3, forkhead box P3. (TIF) [file ppat.1010327.s008.tif]

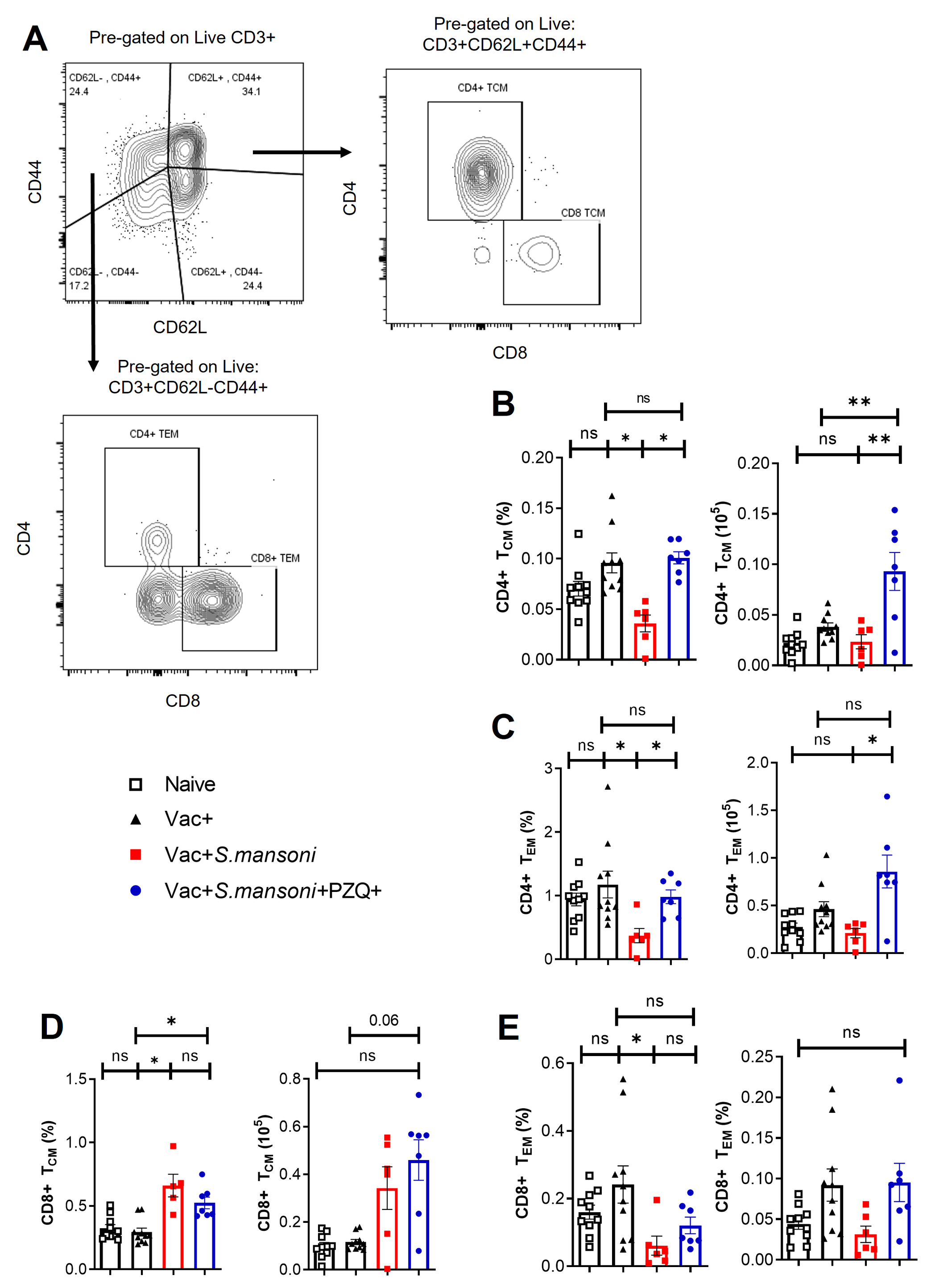

Supplement: S9 Fig — (A) Representative flow cytometry gating strategy for identifying central memory, effector memory and naive T cells. (B) CD3+CD62L+CD44+CD4+ central memory T cells. (C). CD3+CD62L-CD44+CD4+ effector memory T cells. (D) CD3+CD62L+CD44+CD8+ central memory T cells. (E) CD3+CD62L-CD44+CD8+ effector memory T cells. Data are expressed as mean ± S.E.M and representative of 2 independent experiments (n = 6–10 mice per group in each experiment); Data was analysed by the Shapiro-Wilk test followed by either one way ANOVA with Bonferroni’s multiple comparisons test or by Kruskal Wallis followed by the Dunn’s multiple comparisons test; ns, p> 0.05; * p< 0.05, ** p< 0.001, *** p< 0.0001; Vac, hexavalent (DTPa-hepB-IPV-Hib) vaccine; Sm, S. mansoni; PZQ, praziquantel; SSC, Side scatter; CD3, cluster of differentiation 3; CD4, cluster of differentiation 4; CD8, cluster of differentiation 8; TCM, central memory T cells; TEM, effector memory T cells. (TIF) [file ppat.1010327.s009.tif]
