# Supplementary material for: Schistosoma mansoni infection induces plasmablast and plasma cell death in the bone marrow and accelerates the decline of host vaccine responses
Source: PLoS Pathog. 2022 Feb 14;18(2):e1010327. doi: 10.1371/journal.ppat.1010327 (PMC8893680; doi:10.1371/journal.ppat.1010327)
Supplement: S1 Table — (DOCX) [file ppat.1010327.s010.docx]

**S1 Table. Schistosomiasis prevalence: Distribution of children by sex, age groups and *Schistosoma mansoni* infection status.**

| **School** | **Yoro 1** | | **Yoro 2** | | **Bongando** | | **Kedia** | | **Ediolomo** | |
| --- | --- | --- | --- | --- | --- | --- | --- | --- | --- | --- |
| **Total cases** | *N* = 33 | | *N* = 41 | | *N* = 48 | | *N* = 32 | | *N* =35 | |
| **Infection status** | Sm(-) | Sm(+) | Sm(-) | Sm(+) | Sm(-) | Sm(+) | Sm(-) | Sm(+) | Sm(-) | Sm(+) |
| **All  cases,**  **N (%)** | 25 (75.8) | 8 (24.2) | 19 (46.3) | 22 (53.7) | 45 (93.75) | 3 (6,25) | 30 (93,75) | 2 (6.25) | 34 (97.1) | 1 (2.9) |
| **Sex: M/F** | 14/11 | 7/1 | 9/10 | 11/11 | 17/28 | 0/3 | 15/15 | 0/2 | 17/17 | 1/0 |
| **Age groups, N (%)** | | | | | | | | | | |
| **<10 years** | 1 (100) | 0 (0) | 5 (35.7) | 9 (64,3) | 10 (100) | 0 (0) | 7 (87.5) | 1 (12.5) | 8 (100) | 0 (0) |
| **(10 -14) years** | 23 (82.1) | 5 (17,9) | 14 (56) | 11 (44) | 34 (91.9) | 3 (8.1) | 22 (95.7) | 1 (4.3) | 23 (95.8) | 1 (4,2) |
| **>14 years** | 1 (25) | 3 (75) | 0 (0) | 2 (100) | 1 (100) | 0 (0) | 1 (100) | 0 (0) | 3 (100) | 0 (0) |

M, Male, F, Female, Sm, *S. mansoni*
