# Supplementary material for: Schistosoma mansoni infection induces plasmablast and plasma cell death in the bone marrow and accelerates the decline of host vaccine responses
Source: PLoS Pathog. 2022 Feb 14;18(2):e1010327. doi: 10.1371/journal.ppat.1010327 (PMC8893680; doi:10.1371/journal.ppat.1010327)
Supplement: Attachment — Submitted filename: Answers to Reviewers_R1.docx [file ppat.1010327.s011.docx]

**Answers to Reviewers**

Part I - Summary
Please use this section to discuss strengths/weaknesses of study, novelty/significance, general execution and scholarship.
Reviewer #1: In the manuscript the authors investigated the involvement of schistosoma mansoni infection on host anti-poliovirus vaccine response. They suggest that schistosomiasis may induce plasmablast and plasma cell death which limit blood plasma anti-poliovirus specific IgG antibody titer. However, this study is likely to be make less sense. We all know that poliovirus mainly affects children under the age of 6, but the plasma anti-polio IgG antibody titer showed no difference after schistosoma mansoni infection until the children reach the age of 14 in this study. Most important, there are multiple major flaws in study design, especially some critical control groups were not included, and the data that they present is far from supporting their conclusions.

**Poliovirus is a serious, notifiable and highly contagious disease of international significance, even as it is on the verge of eradication globally. Our study adds to the current knowledge that may help the WHO’s drive to eradicate polio (one case is too many cases). Polio high-risk areas usually fall within areas that are known to be endemic for schistosomiasis. In multiple African regions and many others around the globe, there is also a concern for vaccine derived polioviruses (VDPVs), particularly for people who are not able to mount robust immune responses to the live vaccines. These VDPVs can circulate within communities and warrant immediate public health action.**

**The drive to eradicate polio globally is hinged on effective vaccination programs. The WHO recommends targeted mop-up vaccination campaigns to target under protected or unprotected children (**[**https://www.afro.who.int/health-topics/polio**](https://www.afro.who.int/health-topics/polio)**). Given our findings, monitoring the efficacy and hence the effectiveness of polio vaccination in mounting expected immunity in schistosomiasis endemic areas require careful consideration where strategic vaccination policies are concerned. Reduction of polio vaccine immunity in these polio high-risk areas could have dire consequences that may threaten progress in the elimination of the virus globally.**

**Although the reviewer concerns are appreciably noted, it is important to recall that poliovirus can also infect older children who can spread the disease and cause outbreaks through oral faecal contamination (Blake et al., 2014** [**https://dx.doi.org/10.1073%2Fpnas.1323688111**](https://dx.doi.org/10.1073%2Fpnas.1323688111) **). In addition, adults can also suffer from post-polio syndrome and VDPVs. Therefore, the protection of older children and adults is equally important, because these population groups are more mobile hence are likely to facilitate high rates of poliovirus spreading to unrelated areas should they become infected. Older children and adults are also likely to practice poor sanitary behaviours such as urinating and defecating in open spaces (**[**https://doi.org/10.1186/1471-2458-12-140**](https://doi.org/10.1186/1471-2458-12-140)**), thus spreading the virus in schistosomiasis endemic areas which are often high-risk areas for poliovirus infections owing to poor sanitary infrastructure in these remote settings. As such, the importance of robust vaccine memory maintenance even in older cannot be understated.**

**In addition, poliovirus vaccines’ antibody-mediated mode of protection is similar to multiple unrelated viral vaccines. Previous studies including ours (**[**https://doi.org/10.1016/j.pt.2021.07.009**](https://doi.org/10.1016/j.pt.2021.07.009)**) have indicated reduced vaccine-induced antibodies in schistosomiasis infected individuals who are vaccinated for diseases such as measles and hepatitis B. Therefore, the impact of schistosomiasis might not necessarily be centralized to the polio vaccine but viral vaccines with antibodies as correlates of protection. Hence, it is important to identify and report science-backed findings such as ours with the broader scientific community. These data could also be useful in informing PZQ mass administration where catchup mass vaccination is considered in schistosomiasis endemic areas.**

Reviewer #2: The present study is a very good study which is novel and shows important significance.
The present study examined the impact of chronic schistosomiasis on the sustain-ability of vaccine-induced immunity in both children living in endemic areas and experimental infections in mice. Their results demonstrated that showed that S. mansoni infection could be associated with impaired vaccine responses in both children and mice, by lowering the survival of CD138+ plasma B cells as shown in mice, and ultimately suppressing the maintenance of serological memory. This study further demonstrated that PZQ treatment could restore vaccine induced humoral memory responses in S. mansoni infected children, and cellular and humoral immune responses in mice. However, there are minor grammatic errors in the manuscript. After authors revise it, I suggest the manucript be accepted for publishment.

**We thank the reviewer for their thoughtful and encouraging comments and did attend to all minor errors reported by this reviewer below.**

Part II – Major Issues: Key Experiments Required for Acceptance
Please use this section to detail the key new experiments or modifications of existing experiments that should be absolutely required to validate study conclusions.
Generally, there should be no more than 3 such required experiments or major modifications for a "Major Revision" recommendation. If more than 3 experiments are necessary to validate the study conclusions, then you are encouraged to recommend "Reject".
 Reviewer #1: 1. In researches, diagnostic negative only based on single stool examination (Kato Katz) is not reliable. It principally needs to combine with other methods or strategies to efficiently improve the accuracy of diagnosis, e.g. each candidate needs ELISA plus three/five stools for nine/ten detections. However, it seems likes authors did not follow these principals.

**While the reviewer concerns are appreciated, our decision regarding the use of Kato-Katz was an informed one based on the guidelines provided by the World Health Organization (WHO). The WHO traditionally, though still, recommends two Kato-Katz thick smears for accurate diagnosis and quantification of *Schistosoma mansoni* and Soil-transmitted helminth for prevalence mapping and the implementation of control interventions (Montresor A, Crompton DWT, Gyorkos TW, Savioli L, World Health Organization, Montresor A, et al. Helminth Control in School-Age Children: A Guide for Managers of Control Programmes. Geneva: World Health Organization (2002) &** [**https://apps.who.int/iris/bitstream/handle/10665/43545/9241547103_eng.pdf;jsessionid=0F319E2D9941ECB411F67EC69757C5F4?sequence=1**](https://apps.who.int/iris/bitstream/handle/10665/43545/9241547103_eng.pdf;jsessionid=0F319E2D9941ECB411F67EC69757C5F4?sequence=1)**). The Kato-Katz is currently still the gold standard technique for diagnosis (**[**https://apps.who.int/iris/bitstream/handle/10665/42588/WHO?sequence=1**](https://apps.who.int/iris/bitstream/handle/10665/42588/WHO?sequence=1)**). Studies have also shown that 2 to 3 Kato-Katz smears are sufficient for *S. mansoni* diagnosis by Kato-Katz (**[**https://dx.doi.org/10.1371%2Fjournal.pntd.0005953**](https://dx.doi.org/10.1371%2Fjournal.pntd.0005953)**;** [**https://doi.org/10.1371/journal.pntd.0003139**](https://doi.org/10.1371/journal.pntd.0003139) **). To improve sensitivity, two stool samples were collected on two separate days. Briefly, 2 fresh morning stool samples were collected on 2 separate days for each child. For each child, two stool samples per day were assessed by 2 independent laboratory technologists on two different days. Altogether, a total of 4 Kato-Katz smears were assessed (2 slides per sample multiplied by 2). This was indicated in the material and methods section of the revised manuscript with tracked changes (lines 688-694 of the revised manuscript with tracked changes). This recommended and highly specific technique was chosen consistent with the nature of high and persistent transmission area of our study site (**[**https://doi.org/10.1038/s41598-020-59613-z**](https://doi.org/10.1038/s41598-020-59613-z) **;** [**https://doi.org/10.3389/fimmu.2018.02295**](https://doi.org/10.3389/fimmu.2018.02295) **;** [**https://europepmc.org/article/med/12932101#impact**](https://europepmc.org/article/med/12932101#impact) **) while also taking into consideration the technique’s low cost, reliability, skilled expertise, accessibility and logistical constraints such as sample storage and transport in this resource-constrained and remote region of rural Cameroon.**

2. Schistosome antigens (SWA and SEA)-specific antibodies must be used as controls to be detected together with anti-poliovirus antibody, which is critical to clarify their hypothesis.

**Adding to our findings that schistosomiasis infection was associated with reduced blood anti-polio IgG titers in mice over time, further analyses revealed that SEA specific IgG antibodies were elevated over time in the same mice. This points towards the sustenance of schistosomiasis immunity which was not affected as highlighted by exponentially higher SEA titres over time. Treatment of schistosomiasis infected mice at week 10 post-infection resulted in the reduction of SEA IgG antibodies during follow-up at week 2 months post-treatment. Interestingly, the reduction of blood SEA IgG antibodies in treated mice saw a rise in anti-polio IgG antibodies (Figure review 1 below from S4 Figand Fig 2E in the revised manuscript).**

**Figure review 1. Increase in serum SEA IgG titers in over time was associated with reduction of anti-polio vaccine IgG titers in schistosomiasis infected mice. (Graphs extracted from S4I Fig and Fig 2E, respectively)**

**Additionally, these findings in SEA IgG antibodies supported further our observed S. mansoni egg burden (EPG) per gram of liver and gut in polio vaccinated animals. At week 18 post-infection, increases in blood SEA IgG in infected mice matched increases in liver and gut EPGs. Further analyses of schistosomiasis infection on non-vaccinated mice showed comparable levels of bone marrow total B cells, plasmablasts and plasma cells at week 4 post-infection. Indeed, at week 4 post-infection, no eggs were identified in the liver of schistosomiasis infected mice and unsurprisingly there was no liver inflammation as highlighted by similar liver weights relative to mouse body weights in both naïve mice and infected mice. However, slightly and significantly inflamed spleens were observed in infected mice at week 4 highlighting the impact of the parasite on immunological organs early on. Evidence at week 10, however, showed differing results that supported our findings in infected and vaccinated mice. We found significantly reduced plasmablast and plasma cell populations in S. mansoni-infected mice at week 10 post-infection, along with significant liver and spleen inflammation trapped parasite eggs in liver and gut tissues at this time point (S5 Fig in revised manuscript).**

**Figure review 2. Increase in spleen and liver inflammation at week 10 was associated with reduction of bone marrow plasma cells in schistosomiasis infected mice. (Graphs extracted from S5 Fig in revised manuscript)**

**Together, our findings point towards the chronic stage of schistosomiasis pathology as associated with reduced maintenance of B cells, plasmablasts and plasma cells in the bone marrow and this tightly correlates with egg deposition.**

3. A critical control group, Naive+PZQ, was missing in both human and animal experiments. Because PZQ has direct impact on immune system, which may increase B and plasma cells in bone marrow and spleen directly but not indirectly by killing of adult worm. In addition, eggs in host liver and intestine play far important roles in altering host immune responses after infection, while PZQ only efficiently kills adult worm instead of eggs, which further suggests a possibility that PZQ impact on host immune response directly.

**Previous studies have shown that PZQ minimally affect immune responses in the absence of infection. Joseph et al 2004, showed that treatment of whole blood with PZQ in the absence of either SWA or SEA had no significant effect on the levels of IL-4, Il-13, IL-5, IL-10 and IFN-y cytokines (**[**https://doi.org/10.1086/422604**](https://doi.org/10.1086/422604)**). In the presence of SWA and Teg (worm-tegument antigen), Il-5 and IL-13 have been shown to increase after PZQ treatment whereas no significant changes in cytokines were observed for whole blood stimulated by SEA and treated with PZQ. These results signify that PZQ only impacts immune responses in the presence of antigens SWA and TEG. In our study, we think that the PZQ efficiency in killing worms (as shown by reduced egg burden at the experimental endpoint in comparison to non-treated mice) resulted in the curbing of egg production, hence lower numbers of eggs in PZQ treated mice (see Fig 2D) whereas egg production continued in untreated mice. This curbing of egg production meant lower pathology in PZQ treated mice in comparison to untreated mice (as highlighted by reduced hepatosplenomegaly PZQ treated mice, see figures 2B and 2C). Thus, mice with reduced schistosomiasis driven pathology fared better in maintaining immune responses to the vaccine. In support, we found that SEA IgG (S4 Fig) and EPG (figure 2D) in PZQ treated mice was significantly less than that of untreated mice. Hence the significant reduction of anti-polio IgG in infected mice and children being attributable to ongoing schistosomiasis infection and continued egg production in untreated mice.**

**Nevertheless, preliminary evidences in humans do reveal a group of 2 anti-Polio vaccinated individuals initially negative for S. mansoni eggs, who upon follow up did receive PZQ over time (4 treatments starting in 2016 until 2018, see figure review 3 below from fig 1D of the revised manuscript) but became infected (new infection) somewhen as evidenced by screening at the end of the follow up in 2018 with a drastic reduction of their anti-Polio IgG titers. This strongly argues against the sole potential of PZQ to drive the restoration of anti-Polio responses in these hosts but further supports a key role for egg-producing infections in mitigating the anti-Polio vaccine responses.**

**Figure review 3, from fig 1D. Influence of S. mansoni infection and vs PZQ treatment on vaccine-induced anti-poliovirus responses in school children from our study. e. Effect of Schistosomiasis infection, and its treatment with praziquantel, on vaccine-induced plasma anti-poliovirus IgG titres. Sm, S. mansoni; PZQ, praziquantel.**

4. The immune regulation in schistsomiasis is intensively studied but the contribution of plasmablast and plasma cell death, if there is any, to the decreased antibody level is not certain. For example, lower level of anti-poliovirus antibody may also results from schistosome infection-induced Tregs or infection-induced competition of B cell differentiation, which may recover when treat mice with PZQ.

**We agree with the reviewer point that immune regulation in the context of plasmablast and plasma cell death is still a relatively unexplored area. With the well-established impact of Foxp3 expression levels by T cells in driving regulation, we analyzed foxp3 expression intensity in our mouse assays by computing the T cell Foxp3 gMFI (Figure S8D). We found FoxP3 expression in CD3+CD4+ T cells to be significantly higher in the spleen of vaccinated than infected and untreated mice (Vac+Sm+) in comparison to both their treated counterparts (Vac+Sm+PZQ+, p<0.05) and the uninfected vaccinated mice (Vac+, p<0.05) (Figure review 4 below – Figure S8D in manuscript). These results agree with current literature where regulatory T cells are elevated secondary lymphoid organs during the chronic stage of schistosomiasis. As such, vaccine efficacy could be impacted in the spleen if a vaccine is administered during this period. In the bone marrow, however, where the core of the poised antibody-producing plasma cells are found, we found no significant differences in the levels of expression of FoxP3 in CD4+ T cells from all mice groups. Intriguingly, Zaretsky et al 2017 (https://dx.doi.org/10.1016%2Fj.celrep.2017.01.067) suggested that the preservation of FoxP3 expressing CD4 T cells in the bone marrow should prevent the loss of plasma cells in the compartment. In their study, the authors also reported that systemic infections reduce bone marrow plasma cells and bone marrow regulatory T cells. As FoxP3 is lower (although not statistically significant) in the bone marrow of Vac+Sm+ mice in our study, perhaps this reduction also adds to the observed reduced plasma cell and plasmablast populations in schistosomiasis infected and untreated mice. Whatever the case, ultimately, we do not report an expansion of the Foxp3 expression in the T cell compartment in the bone marrow that could support a direct Treg-mediated mitigation of antibody-producing cells and associated functions in this compartment, but these evidences are now added and their contribution better discussed and nuancned in the revised manuscript with tracked changes (lines 410-416; 547 – 555 of the revised manuscript with tracked changes).**

**Figure review 4. Frequencies of FoxP3 (gMFI) expression in CD4+ T cells in the spleen and bone marrow of polio vaccinated mice at week 18 post infection. (Graphs extracted from Figure S8D)**

**With the spleen showing a rise of Foxp3 expression, doubled by the reviewer’s query on the potential competition between B cell subsets rather than death of plasma cells to explain the loss of anti-polio IgG responses in vaccinated mice after schitosoma infection, we did further screen all major B cell subsets in the spleen of our mice (Figure S6 and S7) and report only a reduction of marginal zone B cells (figure S6F) opposing an increase of IgG1 memory B cells (figure S6D) amid a lack of follicular B cell change (Figure S6E) that would altogether not support the reviewer theory of a schistosomiasis-driven alteration of B cell differentiation in favour of antibody production loss from the spleen. These additional analyses have now been added to the revised manuscript to better nuance the conclusion of a tighter link between the reduced production of antibodies and plasma cell death primarily.**

**As such, although circumstancial, the only direct link from our screenings to a reduction in antibody production therefore remained the reduction of bone marrow plasma B cells and plasmablasts. It is therefore important to note the formulation of our results in this respect, where we do not unequivocally claim a causal link between the loss of plasma cells and the drop in antibody responses but do cautiously point out the parallel occurrence of both events to make a suggestive but – still to be further investigated with better tools- claim. It is possible that many different actors are at play here to support the drop in antibody production associated with schistosomiasis and we do extensively report on our observations that do or do not parallel such a reduction, without any conclusive claim of causality or sufficiency.**

5. The single antibody level detection data in Figures 1 & 2 are not strong enough to demonstrate the serological memory of a vaccine. More important mediators and comprehensive indicators are needed, such as memory B lymphocytes, plasma cells, bystander T cells, etc.

**Although the importance of T cells is appreciated, the correlates of protection for the IPV vaccine are anti-polio antibodies measured in blood. Studies have shown that the measurement of blood polio specific antibodies is a suitable method to assess systemic responses to vaccine immunological inductions (**[**https://dx.doi.org/10.1128%2FCVI.00131-10**](https://dx.doi.org/10.1128%2FCVI.00131-10) **;** [**https://doi.org/10.1371/journal.pone.0197370**](https://doi.org/10.1371/journal.pone.0197370)**).**

**Our primary interest, therefore, rested on the influence of schistosomiasis infection and its treatment on these correlates of protection, with particular interest on the bone marrow, a known long-term niche for long term maintenance of antibody-producing plasma cells.**

**However, on this reviewer’s request we further screened B (figure S6 and S7) and T cell (figure S8 and S9) responses in our mouse assays. In the B cell compartment (figure S6), total B cell numbers were drastically reduced in the bone marrow but poorly, if at all, in the spleen by schistosomiasis (vac+sm+ vs vac+ vs naive). Delving deeper into the spleen, we noted that although a minimal elevation of IgG1 memory B cells was observed as a result of vaccination alone (Vac+) when compared to naïve mice, schistosomiasis did not reduce but rather increased the IgG1 memory B cells population in Vac+Sm+ mice and failed to affect the follicular B cell compartment in these mice when compared to vac+ and naïve mice. Strikingly, marginal zone B cells of the spleen were increased by vaccination (vac+), reduced by schistosomiasis (vac+ sm+) to be powerfully restored by PZQ treatment of vaccinated and infected mice (Vac+Sm+PZQ+) making it the only B cell subset in the spleen that parallels the dynamic of anti-polio IgG responses. In the T cell compartment (figure S8/S9), with memory T cells specific to the lymphoid tissue such as the spleen, we noted a vaccination prompted expansion of central memory and effector memory T cells in the vac+ compared to the naïve group which was then reduced following schistosomiasis in Vac+Sm+ to be restored powerfully by PZQ treatment in Vac+Sm+PZQ+ mice paralleling the dynamic of anti-polio IgG responses as well and in support of our used proxy of vaccination efficiency i.e. serum anti-Polio IgG levels. We now add these evidences to our reports emphasizing on the non-exclusive possibility of plasma cell depletion in bone marrow sufficiently driving the drop in antibody production.**

6. Current results in Fig 1 are not sufficient to support conclusion. For example, Fig 1C missed two critical control groups: 1) anti-poliovirus antibody levels from persons without Sm infection at 2016 and 2018 which would help to know whether the antibody level is still in stage of arising. 2) anti-poliovirus antibody levels from PZQ-treated normal person without Sm infection, which may exclude the direct regulation of PZQ on immune system that have been reported in recent years. If there were a group of PZQ+reinfection will be helpful to support their conclusion.

**In figure 1, we do show that a cross sectional screening of vaccinated children do reveal a more lower vaccine response in schistosomiasis-infected children, the figure then shows significant reduction more rapidly over time in schistosomiasis-infected children and then reveal a restoration of vaccine-elicited anti-IgG titers in children susccesfuly treated with PZQ. This altogether is set as a basis to justify further mechanistic studies in mice to question causality in the reduction of vaccine response as a result of schistosomiasis. We therefore respectfully disagree with the reviewer comment of the insufficiency of the figure to support the initial suggestive but quite cautious claims made in the paper.**

**Nevertheless, we did further screened in our cohort data and report additional preliminary data here on children that were negative for Sm and then acquired the disease through several rounds of PZQ treatments over the 3 years period (figure 1D of the revised manuscript). The decay of their anti-polio IgG titers, despite PZQ administration, do strongly argue against the reviewer’s concern and do strongly support our original suggestion of a parasite-specific driven-reduction of vaccine response rather than a sole effect from PZQ administration, a suggestion that led to a series of mouse-based experiments pointing at the same conclusion (reduction of vaccine response in Vac+Sm+ when compared to Vac+).**

**Figure review 5 from figure 1D in the revised manuscript. Influence of S. mansoni infection and vs PZQ treatment on vaccine-induced anti-poliovirus responses in school children from our study. e. Effect of Schistosomiasis infection, and its treatment with praziquantel, on vaccine-induced plasma anti-poliovirus IgG titres. Sm, S. mansoni; PZQ, praziquantel.**

**Screening further our database survey on the site, we found other evidences, though preliminary for the lack of PZQ only effect on anti-Polio IgG titers in vaccinated children (figure review 6 below).**

**Figure review 6. Changes of anti-polio responses relative to schistosoma infection status and PZQ administration.**

**The graph shows a selection process for samples on the left side with 12 children providing exploitable data from 2016 to 2018. The graph on the right shows that :**

- **Anti-polio vaccinated children who went from Sm+ to Sm- had a significant increase of anti-polio titers over time (blue line, n=6)**
- **Anti-polio vaccinated children who went from Sm- to Sm+ had reduced levels of anti-polio IgG over time (red line, n = 2)**
- **Anti-polio vaccinated children who went from Sm- to Sm- show a minimal elevation of anti-polio IgG titers but no decay, though receiving PZQ 4 times (grey line, n=1)**
- **Anti-polio vaccinated children who went from Sm+ to Sm+ showed an absence of polio elevation despite PZQ administration (black line, n=3)**

**The present data are preliminary for most groups (n<4) but do not argue against our central claims that schistosomiasis reduces vaccine responses in our report that led to the in-depth investigations into the murine model – where this was confirmed - in the subsequent part of our study.**

7. The authors tried to conclude the IPV-specific plasmablast and plasma cell apoptosis during schistosome infection, unfortunately, the authors failed to show the “specific”.

**It is first important to note that our report do neither claim apoptosis to be the mean of observed death of plasma cells in the bone marrow here nor does it point at these dying plasma cells and plasmablasts as being polio-specific. We merely report on a general death of plasma cells and plasmablasts in the bone marrow following patent schistosomiasis infection with or without vaccination (new figure S5 added to the revised manuscript showing plasma cell death in infected only animals without vaccination, as shown here in figure review 7).**

**Figure review 7, from figure S5. Sole chronic S mansoni infection depletes bone marrow plasma cells and plasmablasts in mice.**

**This should conclusively argue against any equivocal claim of polio-specific plasma cell death only, as suggested by this reviewer. However, we did perform additional analyses on the correlates of plasma cell changes in the bone marrow of Sm infected animals in the added figure 3G and noted that, dissimilarly, whereas a proxy of schistosomiasis infection i.e. serum titers of anti-SEA IgG do correlate negatively with plasma cell levels in the bone marrow (figure 3G), serum anti-Polio IgG titers rather correlated positively with plasma cell frequencies in the bone barrows of sm-infected mice (figure 3H). This additionally raises a distingo here for plasma cell dynamics relating to schistosomiasis infection vs anti-polio vaccination i.e. schisto reduced their frequencies whereas polio vaccination increases their abundance (see figure review 8 below from figures 3G and 3H of the revised manuscript).**

**Figure review 8 from figure 3: Left - Anti-SEA titres vs bone marrow plasma cell frequencies of schistosomiasis infected Vac+Sm+ and Vac+Sm+PZQ+ mice at week 18 p.i. Right-. Anti-polio IgG titres vs bone marrow plasma cell frequencies of Vac+, Vac+Sm+, and Vac+Sm+PZQ+ mice combined. Data was analysed by Spearman’s correlation.**

8. The results in the manuscript are simply descriptive, mechanisms about how schistosoma mansoni infection induces plasmablast and plasma cell death is not involved at all.

**Regarding the means of cell death that could support the observed reduction of bone marrow plasma cells and plasmablasts in schistosomiasis-diseased mice, we now provide additional analyses of an apoptosis-specific marker, Annexin V, in these plasma cells and plasmablasts from the bone marrow 18 weeks p.i. (figure 5 of the revised manuscript and figure review 9 below).**

**Figure review 9. Apoptosis (Annexin V+ Live/Dead stain-) does not explain plasma cell death in the bone marrow of S mansoni infected mice.**

**It clearly appeared here that apoptosis levels in these cells in the different groups, although present as is to be expected for plasma cells, do not support the differential cell death observed during Sm infection but necrosis (or any other non-apoptotic and membrane-damaging cell death processes) might be additionally at play here. What remains clear is that schistosomiasis infection drive non-specifically cell death of plasma cells and plasmablasts in mice bone marrows.**

More important, they failed to detect the death in any other import immune cell types as controls.

**Other cells types were also screened for cell death in general i.e. B cell subsets i.e. follicular, IgG1 memory and marginal B cell subsets (figure S7) with IgG1 memory B cell death that appeared to be pronounced in the spleen but not paralleled by a reduction of this IgG1 memory B cell type during schistosomiasis (figure S7E).**

**Altogether, inasmuch as multiple processes could be at play here to drive schistosomiasis associated loss of anti-polio IgG antibodies in the serum, the death of plasma cell and plasmablasts associated with the infection in the mice bone marrow are the most robust correlates of immunomodulation here and soberly reported as such.**

In addition, authors only used a method which is impossible to distinguish the types of cell death.

**Regarding the means of cell death that could support the observed reduction of bone marrow plasma cells and plasmablasts in schistosomiasis-diseased mice, we now provide additional analyses of an apoptosis-specific marker, Annexin V, in these plasma cells and plasmablasts from the bone marrow 18 weeks p.i. (figure 5 of the revised manuscript and figure review 10 below).**

**Figure review 10 from figure 5. Apoptosis (Annexin V+ Live/Dead stain-) does not explain plasma cell death in the bone marrow of S mansoni infected mice.**

**It clearly appeared here that apoptosis levels in these cells in the different groups, although present as is to be expected for plasma cells, do not support the differential cell death observed during Sm infection but necrosis (or any other non-apoptotic and membrane-damaging cell death processes) might be additionally at play here. What remains clear is that schistosomiasis infection drive non-specifically cell death of plasma cells and plasmablasts in mice bone marrows.**

9. Schistosomes are very special worms that quite different from other helminths. Schistosomes lives in blood vessels, which allows the worm to release excreted secretions directly into the blood. And some of the secretions are toxic to the human cells that may include plasmablast and plasma cell. However, it is most likely that this will not happen when hosts infect with other helminths. So, it is not accurate for the authors to state “to uncover the immunological interactions between the vaccine-elicited responses and helminth infection” in line 92. As a better choice, the patients listed in exclusion criteria (Lines 116-118), especially patients co-infected with geohelminths, could be set as control groups.

**Our paper investigates the impact of schistosomiasis on vaccine immunity and does not intend to make assertions about other helminths. We have therefore amended line 92 to reflect this, as ‘**to uncover the immunological interactions between the vaccine-elicited responses and schistosomiasis infection**’. See lines 100-101 of the revised manuscript with tracked changes.**

Reviewer #2: No.

Part III – Minor Issues: Editorial and Data Presentation Modifications
Please use this section for editorial suggestions as well as relatively minor modifications of existing data that would enhance clarity.

Reviewer #1: 1. Line 142, “impactserological” should be “impact serological”. Line 250, “restoredvaccine-induced” should be “restored vaccine-induced”. Line 267, “cellsin” should be “cells in”. 2.

**All corrected as requested.**

Fig 2. This figure should be moved to supplementary material.

**Figure 2 was moved to supplementary material as figure S2.**

What solution or vesicle was used in the mock injection group? “Naïve” should be “Mock”?

**Naïve groups were first treated with a control solution of 1 x PBS to equate the carrier solution of the Hexaxim vaccine for the comparison Naïve vs Vac+. For the next steps, this group was mock infected by percutaneous exposure to Cercariae snail water and then mock fed with 1 x PBS and sterile water containing (10 parts 70% Tween + 30% Ethanol) to equate the carrier solution in which PZQ was diluted. As such this group does not constitue the general mock-control for all groups i.e. naïve is a mock control of vaccination for the group vac+, but vac+ is the mock control for infection for the group vac+sm+ and vac+sm+ is the PZQ treatment mock control for Vac+Sm+PZQ+. What remains clear is that the naïve group is unequivocally a naïve group having no vaccination, no infection and no PZQ treatment. To avoid ambiguity in referring to this group alone as mock control we have resorted to maintain the present appellation of naïve group that applies to all comparison made and did provide extensive definition of the groups throughout the revised manuscript with tracked changes (see S3 Fig and legend).**

**Indeed no response specific to the vaccine was observed in this naïve group, which is not used as a reference to define the impact of schistosomiasis on vaccination. Whatever the case, we also do provide an abridged infection scheme where naïve mice are compared to Sm-infected mice to assess the impact of infection without any influence from the vaccine solution (S5 Fig).**

3. Fig 3. 1) 3A-3D suggest serious pathogenesis in mice livers and intestines in group of Vac+Sm without PZQ treatment, which may significantly impact on the synthesis of antibodies. Thus, Schistosome antigens (SWA and SEA)-specific antibodies must be used as controls.

**We have now included SEA IgG antibodies kinetics over the infection period. While SEA IgG antibodies increased in infected only mice, PZQ treated mice had reduced SEA IgG antibodies post-treatment at the experimental endpoint (figure S8). Additional analyses in mice have shown a significant inverse correlation between liver and gut Sm+ egg burdens to anti-polio IgG antibody titers. Furthermore, our analyses of week 4 infection (figure S5) show no marked differences in liver pathology in comparison to non-infected mice. Week 4 is generally known to correspond to the acute phase of schistosomiasis. Indeed, although the liver is without inflammation, we observed inflammation in the spleens of infected mice at week 4. These results indicate that infection was in its acute phase at week 4. At week 10, liver inflammation was observed along with S. mansoni egg isolation from the liver, showing the chronicity of the infection. Spleen and liver inflammation at this stage were more apparent in comparison to both naïve mice and week 4 infected mice. At week 4 where spleen pathology was minimal and no liver pathology was noted, bone marrow plasmablasts and plasma levels were similar to their non-infected littermate controls. However, at week 10 where splenomegaly and hepatomegaly were more apparent along with parasite egg depositions in the liver (the chronic stage), plasmablasts and plasma cells populations were lower in infected mice when compared to their non-infected counterparts. Combined with elevated SEA IgG antibodies in schistosomiasis infected mice as shown for vaccinated mice, it is agreeable that schistosomiasis in its chronic phase reduces non-specifically the maintenance of plasmablast and plasma cells in the bone marrow. In fact, we do now provide parasite specific antibody responses in the form of SEA-IgG (figure S4 of the revised manuscript).**

2) 3F, the author state that “PZQ treatment significantly restored vaccine-induced IgG antibody titres”, however, the antibody titre of PZQ treatment group is still significantly much lower than that of Vac+ group, so what is the effect of this restoration?

**We thank the reviewer for their comment. Given the non exhaustive assessment of PZQ restorative ability here, we do believe the partial restoration observed is sufficient to make the present claim both in Humans and mice. To sober up our conclusions, nevertheless, we have now added the clarification of a partial restoration throughout the text. Clearly a dose dependent and time-dependent assessment of the effect of PZQ restoration could better tell whether a total restoration will be achievable but do not counter the claims made here of an ameliorated response following PZQ treatment of Schistosomiasis-diseased hosts in humans and mice.**

4. Fig 4. 1) A critical control group, Naiver+PZQ, was missing, because PZQ has direct impact on immune system, which may increase B and plasma cells in bone marrow and spleen directly but not indirectly by killing of adult worm. In addition, eggs in host liver and intestine play far important roles in altering host immune responses after infection, while PZQ only efficiently kills adult worm instead of eggs, which further suggests a possibility that PZQ impact on host immune response directly.

2) Two important control groups, Sm and Sm+PZQ, were missing.

**We thank the reviewer for their comment. The impact of Sm+ in the acute and chronic phase was shown in our supplementary data in mice at week 4 and week 10 (figure S5). Additional supplementary findings on liver and spleen pathology at both time points have been added (figure S5). Briefly, schistosomiasis did not affect the expression of plasmablasts and plasma cells at week 4 (acute phase) whereas plasmablasts and plasma cells were reduced in schistosomiasis diseased mice at week 10 post-infection (chronic stage).**

**Moreover the screening in humans do further help cover all these potential groups, though preliminary, but in great support of our central message here.**

**In fact, Screening our database survey on the site, we found further evidences, though preliminary for the lack of PZQ only effect on anti-Polio IgG titers in vaccinated children (figure review 11 below).**

**Figure review 11 = figure review 6. Changes of anti-polio responses relative to schistosoma infection status and PZQ administration.**

**The graph shows a selection process for samples on the left side with 12 children providing exploitable data from 2016 to 2018. The graph on the right shows that :**

- **Anti-polio vaccinated children who went from Sm+ to Sm- had a significant increase of anti-polio titers over time (blue line, n=6)**
- **Anti-polio vaccinated children who went from Sm- to Sm+ had reduced levels of anti-polio IgG over time (red line, n = 2)**
- **Anti-polio vaccinated children who went from Sm- to Sm- show a minimal elevation of anti-polio IgG titers but no decay, though receiving PZQ 4 times (grey line, n=1)**
- **Anti-polio vaccinated children who went from Sm+ to Sm+ showed an absence of polio elevation despite PZQ administration (black line, n=3)**

**The present data are preliminary for most groups (n<4) but do not argue against our central claims that schistosomiasis reduces vaccine responses in our report that led to the in-depth investigations into the murine model – where this was confirmed - in the subsequent part of our study.**

3) 4B vs 4C, why vaccination only increased B cells in bone marrow but not in spleen?

**Vaccination did also drive B cell increase in the spleen. This is masked here by the multi-group statistics used here (ANOVA with correction) instead of a one-to-one comparison between the naïve and the vaccinated groups (Mann-WHITNEY/T-test). A zoomed representation of these 2 groups naïve vs Vac+ is as follows:**

**Figure review 12 from figure 3C: Higher stringency of multigroup comparison vs one-by-one comparison for two naïve vs Vac+ groups**

**In our represented comparison, we have used ANOVA / Kruskal Wallis with posthoc analyses. These analyses tend to be more stringent in computing the statistical significance where multiple groups are compared. However, the naïve group in this instance is solely to assess the impact of vaccination of cell populations in vaccinated mice, which in turn are compared to the Sm+ infected and treated mice to assess the impact of schistosomiasis and its treatment on vaccine-induced immunity. Between naïve and Vac+, p=0.065 for ANOVA but p = 0.034 for t-test.**

4) 4B\4C\4D, there is no increase of B and plasma cells either in bone marrow or spleen after Sm infection (actually the exact group was missing), thus, how antibodies comes?

**Figures S5 and 3 of the revised manuscript show that B cells and plasma cells are reduced in the bone marrow of schistosomiasis infected mice very late on. This is shown for week 18 (Figure 3) and Week 10 (Figure S5D-F) post schistosomiasis infection (chronic stage). On the other hand, in a new set of experiments involving naïve vs Sm infected mice only (no vaccination, no PZQ treatment in figure S5 A-C), bone marrow B cells are shown elevated in the acute phase of the infection as early as week 4 post-infection (Figure S5A-C) which would justify the antibody production as a result of s mansoni infection (Figure S4). However, late schistosomiasis infection when chronicity sets in do lead to a massive drop in B cell subsets within the bone marrow (figure S5 D-F), supporting at later stage a more immunomodulatory but clearly selective effect of the infection on the antibody-producing machinery.**

5) 4F, results showed there is no significant CD138+plasma cells were induced in Vac+ group, how antibodies comes?

**Former figure 4F, now 3F shows plasma cells in the spleen at the experimental endpoint. Plasma cells are known to migrate from the spleen to bone marrow niches where they survive for long periods as long-term antibody producing cells. Hence, upon vaccination 18 weeks before the experimental endpoint, the plasma cell numbers are not expected to remain high in the spleen but are expected to migrate and reside in the bone marrow where they continuously produce vaccine-specific antibodies (this is indeed what is observed in Figure 4D and consistent with the literature (**[**https://doi.org/10.1128/jvi.69.3.1895-1902.1995**](https://doi.org/10.1128/jvi.69.3.1895-1902.1995)**). As such, memory B cells rather than plasma cells would be expected to be elevated in the spleen of vaccinated mice. Indeed, our results show that class-switched IgG1 memory B cells remained resident at significantly higher levels in the spleens of vaccinated mice when directly compared to non-vaccinated mice at week 16 and week 19 post-vaccination (see figure review 13 below).**

**Figure review 13. Vaccine elicited spleen IgG1 memory B cells at week 18 post vaccination (Figures adapted from Figure S2D and Figure S6D). Note that S4D statistics in a one-on-one comparison is more sensitive and unveil massive significance (p<0.001) as opposed to multigroup-comparison by ANOVA (p=0.087)**

6) 4F, the percentage and number of the cells in PZQ treatment group are higher than or similar to those of Vac+ group, but the antibody titre in PZQ treatment group is significantly lower than that of Vac+ group (eg. Fig 3E). These results are contradictory.

**Plasma cells are not stored in the spleen but bone marrow for long term memory humoral responses. Former figure 4F now 3F shows plasma cells in the spleen and figure 3C shows total B cells in the spleen at the experimental endpoint (>18 weks after vaccination), by which stage vaccine-induced plasma cells are expected to reside in the bone marrow for long term survival and poised to sustain long-term production. Class switched memory B cells rather than plasma cells would be expected to be elevated in the spleen of vaccinated mice as we have shown (figure S6D and figure review 13 above). Figure 2E shows vaccine-specific antibodies from the blood. Long term antibodies in the blood are known to be produced by plasma cells in the bone marrow. Therefore, a direct comparison of spleen plasma cells or B cells and bone marrow produced antibodies at week 19 post-vaccination would potentially be misleading. Moreover, the expectation of a direct translation from B cell numbers to antibody production by the reviewer is overly optimistic as we do cautiously reasoned here that , even for plasma cells in the bone marrow the implication in the antibody production observed is not sufficient but might act in concert with other processes to translate into the parasite-driven reduction of antibody titers observed. We merely provide here a potentially contributing actor to the phenotype observed.**

5. Fig 5.1) Two important control groups, Sm and Sm+PZQ, were missing.

**The Sm+ only group results are now part of the supplementary study at week 4 and week 10 (figure S5) and analyzed in depth for plasma cell dynamics. Our results at week 10 post-infection in these analyses (figure S5) do show the impact of schistosomiasis in the chronic stage of the disease. As our experimental endpoint of week 18 post-infection is schistosomiasis still in its chronic phase, we ended the confirmatory experiment at week 10 in accordance with the internationally established ARRIVE guidelines on animal experiments to minimize and avoid the unnecessary suffering of research mice. Our results at week 18 and at week 10 (both in their chronic forms) confirmed the depletion of B cells and plasma cells in the bone marrow of schistosomiasis infected mice.**

**For a group Sm+PZQ, although redundant here in mice (gleaned from the comparison between vac+ Sm+ and Vac+ Sm+PZQ+), we provide to the attention of this reviewer preliminary data from the field on humans. Screening our database survey on the site, we found further evidences, though preliminary for the lack of PZQ only effect on anti-Polio IgG titers in vaccinated children (figure review 14 below).**

**Figure review 14. Changes of anti-polio responses relative to schistosoma infection status and PZQ administration.**

**The graph shows a selection process for samples on the left side with 12 children providing exploitable data from 2016 to 2018. The graph on the right shows that :**

- **Anti-polio vaccinated children who went from Sm+ to Sm- had a significant increase of anti-polio titers over time (blue line, n=6)**
- **Anti-polio vaccinated children who went from Sm- to Sm+ had reduced levels of anti-polio IgG over time (red line, n = 2)**
- **Anti-polio vaccinated children who went from Sm- to Sm- show a minimal elevation of anti-polio IgG titers but no decay, though receiving PZQ 4 times (grey line, n=1)**
- **Anti-polio vaccinated children who went from Sm+ to Sm+ showed an absence of polio elevation despite PZQ administration (black line, n=3)**

**The present data are preliminary for most groups (n<4) but do not argue against our central claims that schistosomiasis reduces vaccine responses in our report that led to the in-depth investigations into the murine model – where this was confirmed - in the subsequent part of our study.**

2) 5B and 5C showed that the mock/naïve treatment resulted in the biggest numbers of dead plasmablast and plasma cells in bone marrow, however Vac, Vac+Sm, and Vac+Sm+PZQ saved the cells from death?

**Vaccination rescue plasma cell from death to mediate the generation and persistence of long-lived plasma cells. The generation of these protective antibody-secreting cells can be triggered by an exposure to pathogens, and also by vaccination (reviewed in Front. Immunol., 24 April 2019 |** [**https://doi.org/10.3389/fimmu.2019.00788**](https://doi.org/10.3389/fimmu.2019.00788) **). This what is observed here where the naïve, non-challenged and non-vaccinated group shows a high rate of plasma cell death, as expected in the absence of vaccine stimulus. The vac+ group do rescue such cell death and foster plasma cell survival. Sm+Vac+ group then reveals a loss of the protective advantage of vaccination on plasma cell survival as a result of schistosomiasis infection. Finally, Vac+Sm+PZQ+ shows that PZQ treatment after loss of the protective advantage of Vac+ by Sm+ on plasma cell survival is now significantly restored by PZQ treatment i.e.. Sm removal.**

3) Does the death of antibody-producing plasma B cells includes schistosome antigen-specific antibody-producing plasma B cells? What about paralleled levels of schistosome antigen-specific antibodies?

**We have added supplementary data with SEA IgG antibody kinetics to our findings (figure S4 of the revised manuscript). We have also added supplementary data correlating anti-polio IgG to *S. mansoni* egg burden at the experimental endpoint (week 18 post-infection). Our results show that despite reductions in B cells and plasma cells in the bone marrow, SEA IgG levels were elevated in both Vac+Sm+ and Vac+Sm+PZQ+ mice, although Vac+Sm+PZQ+ mice had significantly less SEA IgG antibodies in comparison to their non-treated counterparts Vac+Sm+ (certainly as a result of Sm clearance by the drug in Vac+Sm+PZQ+).**

6.Discussion.
1) What type of cell death is of interesting, e.g. apoptosis, necrosis, pyroptosis, or ferroptosis? Does the generation and/or differentiation of cells contribute to the decrease? These issues needs to be discussed.

**The type of cell death in our study, although unknown from our assays, was now discussed and the added possibility of the contribution of subset differentiation explored. See lines 533-577 of the revised manuscript.**

2) Line 386. Without detection of any other antibodies, current data in the manuscript is not enough to support the conclusion like “……IPV specific serological memory……”. Similarly, all words of “specific” in the manuscript need to be carefully evaluated.

**While we are thankful to the reviewer for their comment and suggestion, we respectfully disagree with the reviewer because the highlight regarding IPV specific antibodies is correct because our antibody assays were specifically designed for the detection of polio antigens thus anti-polio specific IgG responses (Human Anti-Poliomyelitis Virus 1-3 IgG ELISA Kit, Alpha Diagnostic International, Tx, USA and Mouse Anti-Poliovirus Virus 1-3 IgG ELISA Kit, Alpha Diagnostic International, Tx, USA). However, references to polio-specific plasma cells or plasmablasts or polio-specific IgG1 memory B cells were not found nor included in the entire text as we do not present nor claim characterization of these cells as such a level of specificity in this study.**

Reviewer #2: There are minor grammatic errors in the manuscript.

**The manuscript was carefully scrutinized and grammatical errors found were corrected.**
