# Supplementary material for: Schistosoma mansoni infection induces plasmablast and plasma cell death in the bone marrow and accelerates the decline of host vaccine responses
Source: PLoS Pathog. 2022 Feb 14;18(2):e1010327. doi: 10.1371/journal.ppat.1010327 (PMC8893680; doi:10.1371/journal.ppat.1010327)
Supplement: Attachment — Submitted filename: Answers to Reviewers_R2.docx [file ppat.1010327.s012.docx]

**Answers to Reviewers**

Reviewer #1: 1. The title of the manuscript that "Schistosomiasis induces plasmablast and plasma cell death in the bone marrow and accelerates the decline of host vaccine responses". It is inappropriate and should be changed to: “Schistosoma mansoni infection induces plasmablast and plasma cell death in the bone marrow and accelerates the decline of host vaccine responses”.

**The manuscript title has been changed, as recommended.**

2. Sample size in each of the experiments should be explicitly mentioned throughout the results and should be included (n=) in the figure legends as much as possible. This is crucial to assess the robustness of the findings and put in perspective statistical significance.

**The sample size, n, has been added to all figure legends where appropriate.**

3. The discussion seems messy and it should be even more concise.

**We thank the reviewer for their comment and suggestion. The discussion has been extensively reviewed and substantially improved, making sure all key aspects of the findings are comprehensively but cautiously interpreted to leave no room to unsubstantiated conclusions.**

4. Also, there are still a number of language and typo errors throughout. I would still recommend revise language throughout the manuscript.

e.g.,

line 99: a S. mansoni

line 101: schistosomiasis infection

line 249: at week 4, 8, and 18 p.i. ……

line 793: using the the two-stage

etc.

**The manuscript has now been extensively reviewed by a native English language speaker to improve the language and typos were corrected.**
